# Dealing With Environmental Stress: Why Is It Time to Move Towards a New Approach to Depression?

**DOI:** 10.7759/cureus.74566

**Published:** 2024-11-27

**Authors:** Claudio Russo

**Affiliations:** 1 Health and Technology, Stress Management and Therapy Clinic, Naples, ITA

**Keywords:** depression, healthy education environment, medicaid barriers to telehealth, mental health and endocrine diseases, neuro-immunology

## Abstract

Depression is a complex mental health condition characterized by persistent sadness, loss of interest, and a range of cognitive, emotional, and behavioral symptoms. It can be acute or chronic and is often influenced by a combination of genetic, biological, psychological, and social factors. According to transnational estimates of prevalence, depressive symptoms represent the most concerning challenge to mental health across cultures and beyond geographical borders. There are growing public health concerns that depression is leading to an increase in suicide rates, while causing diversity, uncertainty of treatment options and cultural stigma. The current trends in life expectancy make depression a great challenge for global health, while there are unsolved care problems on how to implement adequate responses and treatment at local and global levels. The main purpose of this article is to highlight why an integrated path to care is more critical than ever before. The role of biomedical sciences and modern psychology is therefore discussed in terms of how developing our knowledge may better help us to support those with depression, and to inspire new research methods in primary and secondary care.

## Editorial

Background

The healthcare management of chronic illness is one of the major public health challenges. Multifaceted risk factors, individual vulnerabilities and areas of social intervention have been extensively taken into account when treating mental health conditions, including anxiety and depression. Overall, depressive disorders have been accounted as the major contributor to non-fatal health loss since 2017, with 322 million cases recorded in the world, equal to 4.4% of the global population [1]. In general, the current approach to care confirms that depressive disorders are the major mental health condition in mental health illnesses across the globe. This primary factor could contribute to increased public health expenditure, particularly when considering the potential cost-effectiveness of an integrated care approach. Specifically, medical prescriptions and effective health management strategies are raising concerns about how public health budgets should be allocated to provide adequate healthcare and improve patient outcomes.

This article outlines an ethical model for mental health care and explores strategies for developing integrated care pathways for depression. Throughout the adoption of multidisciplinary efforts, suggestions are made on how to use a different approach to mental health care that can prove to be useful in the diagnosis and treatment of depressive disorders. The environment, according to Valsiner’s perspective of 'co-genetic logic,' refers to the processes by which individuals attribute individual signs to their experiences, thereby producing mediated representations that help interpret thoughts and feelings. Co-genetic logic emphasizes how these signs are co-created through the interaction between a person and their environment [2]. In depression, a specific attribution to symptoms will cause feelings of sadness, loss of interest, and social withdrawal. Environmental stress can be considered a precipitating factor for depressive conditions, leading to non-adaptive physiological responses, maladaptive psychological reactions, and dysfunctional social interactions.

Identifying markers in depression

**Figure 1:**
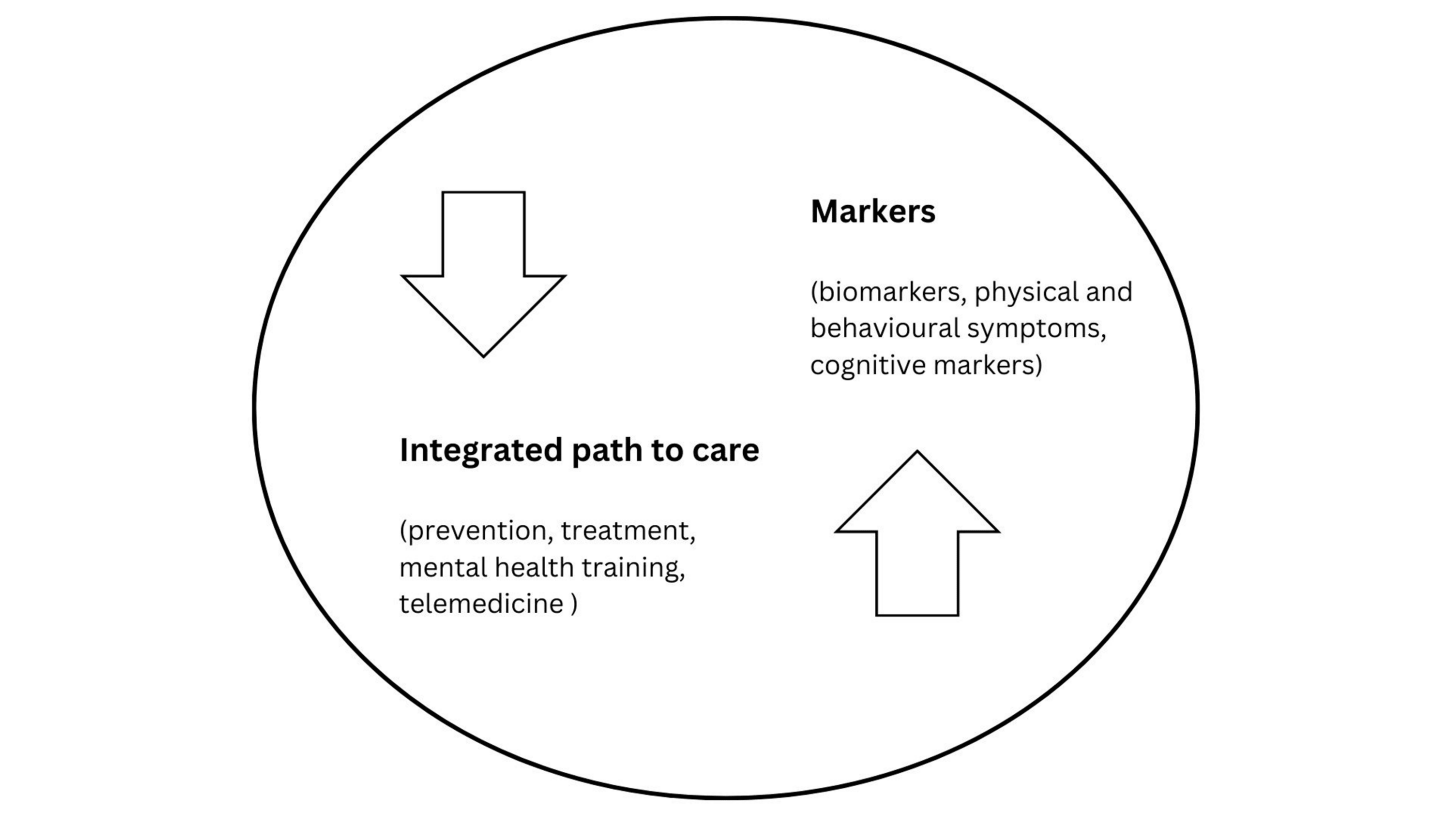
From markers to an integrated path to care of depressive symptoms Original work: copyright is retained by the author.

To identify shared risk factors with non-communicable diseases (NCDs) and mental health, it is suggested that substance abuse, unhealthy diet, environmental changes and other medical conditions are accounted for the development of illness [3]. Indeed, the clinical management of depression needs to be assessed along with the co-existence of other diseases. That being said, the main goal for professionals is to improve a person's adherence to treatment, while supporting and encouraging patients to co-develop an integrated path to care (primary objective; Figure 1).

**Figure 2:**
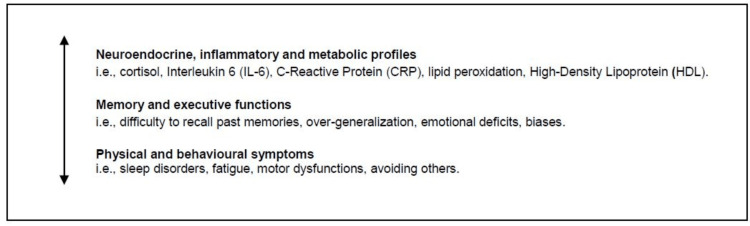
The study of connectivity and biomarkers Original work: copyright is retained by the author.

The analysis of biomarkers should take into account depressive symptoms as part of large interconnected networks, which include the study of connectivity and clinical manifestations of physical, cognitive and behavioural symptoms. Integrating biomarkers and connectivity in the assessment of depression should nurture the clinical objective of examining etiology and treatment of the disorder, namely, a variable expression of depressive symptoms across the lifespan, and the individual diversity to produce the desired outcome and a favorable prognosis [4]. An increasing body of literature suggests the possible biomarkers for oxidative stress [5], inflammation, metabolic profiles, and the role of neurotransmitters [6]. Similarly, a systematic observation of connectivity among the patients (i.e., the relations between biological profiles and individual signs or symptoms) is crucial to provide continuous professional development and practical guidelines in hospital settings (Figure 2).

Telehealth and emerging technologies have the potential to enhance the diagnosis, treatment, and management of depression by facilitating personalized care based on individual patient symptoms and needs. This logical approach is more useful to reach the main goal of developing and integrating tailored models of diagnosis and treatment. On the other hand, cognitive therapies should consider the ever-evolving knowledge on executive functions and memory, and its impact on the behavioural management of other physical symptoms [7].

Advances in clinical practice and depression research

The following directions are capable of driving future experimentation in depression research, moving beyond pre-existing parameters that are important to the clinical practice, and guiding mental health research on depression-related conditions towards a review of ethics-based care management of either acute or chronic conditions. A list of five principles is hereby recommended for professionals to guide them in clinical research:

1. Undertake systematic literature reviews and multidisciplinary efforts to detect shared risk factors and environmental determinants in the early onset of mental disorders and other medical conditions.

2. Integrate a developmental and evolutionary framework based on biological and cognitive-behavioural markers, and adapt existing healthcare services of diagnosis and treatment for depressive disorders.

3. Implement psychological models focusing on cognitive training, emotional management, and social support strategies to encourage health professionals to engage in active listening with service users, within the framework of a collaborative, user-focused care team.

4. Use digital technologies to ameliorate the remote monitoring strategies for mental health conditions, collect physical parameter data and consider the environmental determinants on an individual case-by-case basis.

5. Understand cultural aspects of depression and building new models of care.

Implications

Depressive symptoms differ on an individual level across ages, genders and borders. Similarities or differences refer to the onset and severity of illness, comorbidity with other conditions, and the treatment options that should be tailored and adapted to specific care settings. Indeed, healthcare professionals should address the health priorities under given clinical circumstances, and adapt healthcare plans to every patient. Depression can be either persistent or reoccurring, whereby its clinical manifestations could, in some cases, lead to functional impairments in family and social contexts throughout the lifespan of a person. The complexity of one's living environment can lead to limitations in school achievements or impair work capabilities. In most severe forms, depressive symptoms include self-harm thoughts, significant limitations to everyday activities, as well as suicide risks [8]. In depression, environmental risk factors include housing needs, educational opportunities, employment-related issues, and adverse childhood experiences [9], in addition to demographics and a broad range of social determinants [10].

From this perspective, healthcare professionals can collaborate with patients to enhance preventive and promotional health efforts. This involves implementing new screening procedures in primary care and adapting evidence-based interventions to community-based care settings. Such an approach is crucial for mitigating the effects of chronic depressive illness and reducing symptom relapse (Table 1).

**Table 1:** A new comprehensive approach to build models for treating depression

Demographic/social data	Markers	Environmental risks factors	Unhealthy conditions
Age/gender/civil registrar	Neurotransmitters	Housing	Substance abuse
Education and qualifications	Metabolic profiles/inflammation	School/employment	Unhealthy diet
Geographical area	Memory and executive functions	Family and community	Physical inactivity
Access to health care	Physical and behavioural symptoms	Childhood adversities	Other medical conditions

Addressing public health implications of depression care is crucial to support health professionals and enable them to assess the possible relations between the frequency of markers, long-term resolution of symptoms, and patient outcomes. This is suggested to guide future clinical research, as well as healthcare professionals and, more generally, all stakeholders. In general, the new frontiers in depression research can help individuals feel better on a long-term basis, and at the same time help healthcare providers overcome professional barriers that are currently limiting a new scientific understanding of depression, or hindering the multidisciplinary collaboration across all levels.
